# Supplementary material for: How measurement noise limits the accuracy of brain-behaviour predictions
Source: Nat Commun. 2024 Dec 12;15:10678. doi: 10.1038/s41467-024-54022-6 (PMC11638260; doi:10.1038/s41467-024-54022-6)
Supplement: Supplementary file 1 — Supplementary Information [file 41467_2024_54022_MOESM1_ESM.pdf]

Supplementary Results

Prediction accuracy for age prediction - additional metrics

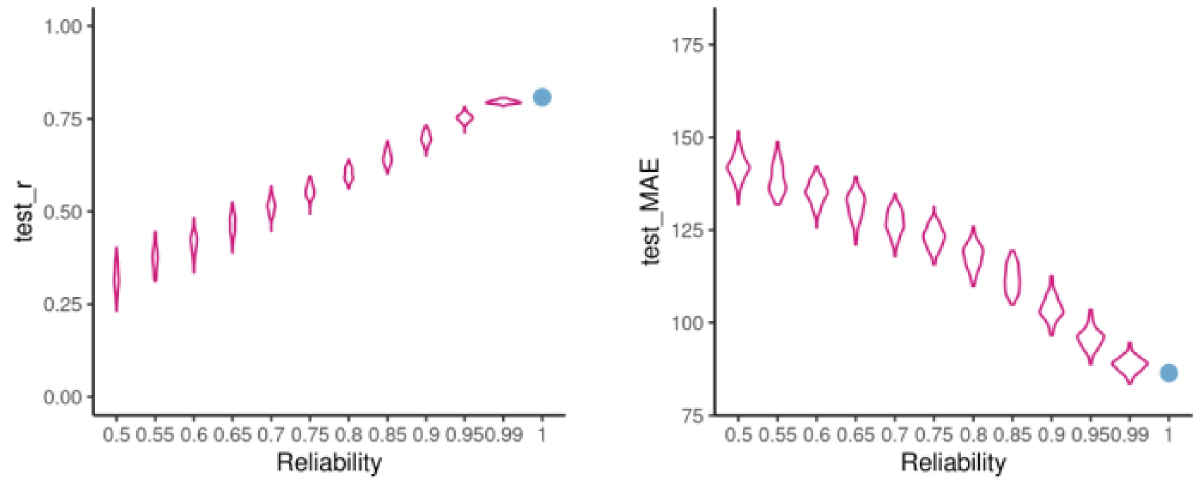

Supplementary Figure 1. Mean absolute error (MAE) and correlation between observed and predicted targets in age prediction. Each violin plot summarises the accuracy of predicting 100 simulated datasets within each reliability band.

Sensitivity analyses - Age prediction

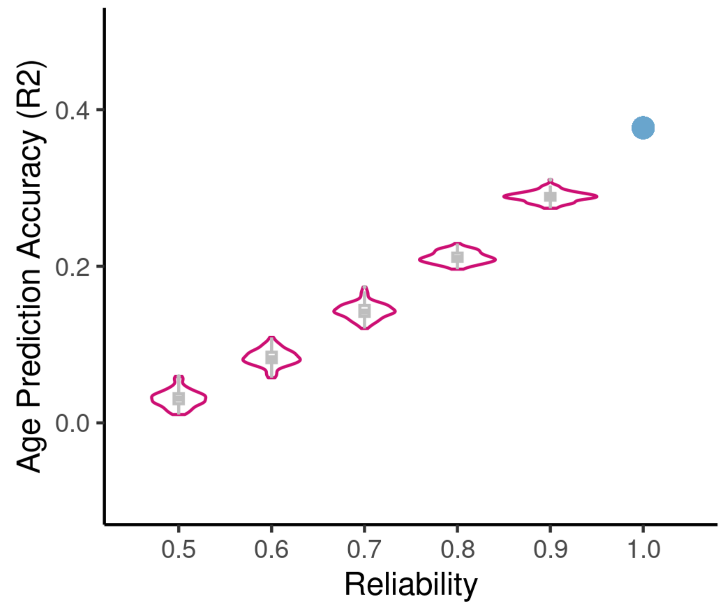

Supplementary Figure 2. Prediction of age using 5000 subjects from the UKB. The same sample of participants was used to create this figure as in the section “Behavioural reliability

is related to prediction accuracy” of the results. The age of subjects was predicted using ridge regression as in Figure 1 and accuracy was evaluated using R2. Each violin plot summarises the accuracy of predicting 100 simulated datasets within each reliability band. Boxplots are centred at the median, with the bounds representing the interquartile range, and whiskers the min/max values, outliers are not shown.

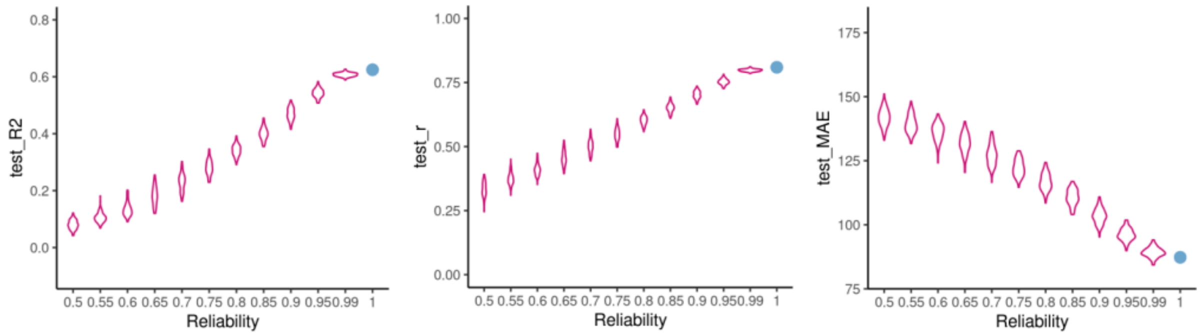

Supplementary Figure 3. Prediction Using Seitzman et al. (2020) Nodes. R2, Mean absolute error (MAE) and correlation between observed and predicted targets in age prediction using 300 nodes by Seitzmann et al. 2020. Each violin plot summarises the accuracy of predicting 100 simulated datasets within each reliability band.

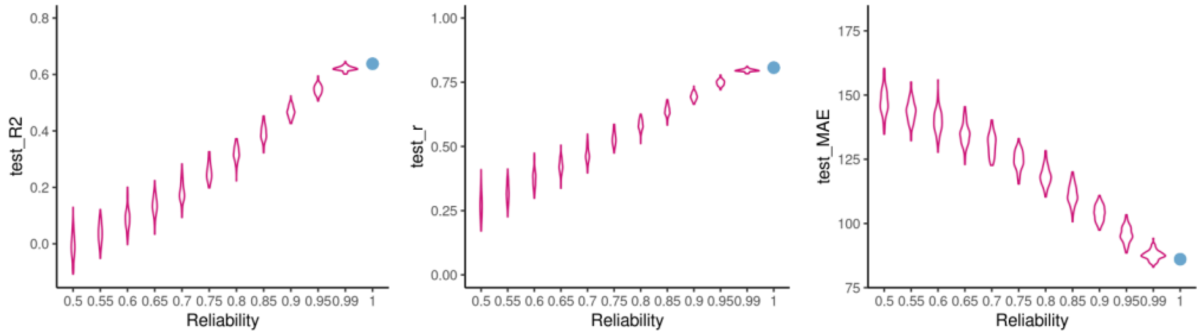

Supplementary Figure 4. Prediction Using Support Vector Regression. R2, Mean absolute error (MAE) and correlation between observed and predicted targets in replication of age prediction using support vector regression. Each violin plot summarises the accuracy of predicting 100 simulated datasets within each reliability band.

Impact of reliability on prediction accuracy in the HCP-A dataset - influence of connectivity reliability

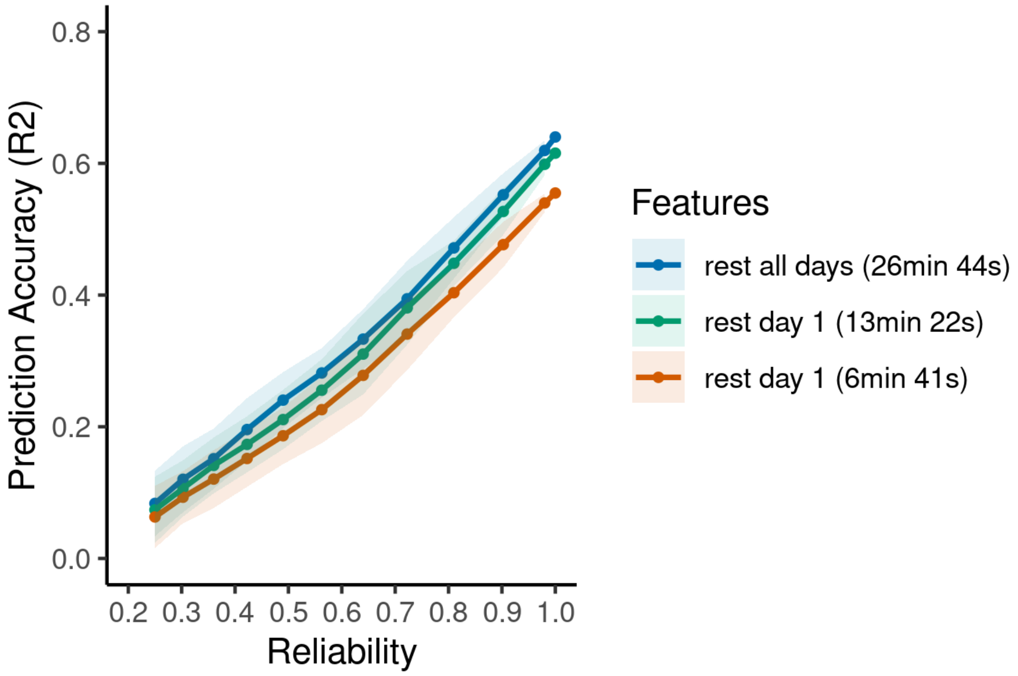

Supplementary Figure 5. Influence of feature reliability (functional connectivity) on the prediction of age with decreasing reliability. The x-axis represents the reliability of age, starting with empirically measured age at ICC ≈ 1.0, followed by age with reduced reliability through simulations as in the main text. Feature reliability (represented by colours) was manipulated by reducing the amount of rsfMRI time course used to calculate functional connectivity, from the average of all four sessions collected on both days used in all main analyses to an average of 13 minutes collected on the first day only and finally, connectivity calculated from a single 6 minutes session collected on the first day in the anterior-to-posterior direction (same as only session in the UKB). Solid lines represent the mean prediction accuracy across all 100 simulated datasets in each reliability band, shaded areas represent 2 standard deviations in prediction accuracies.

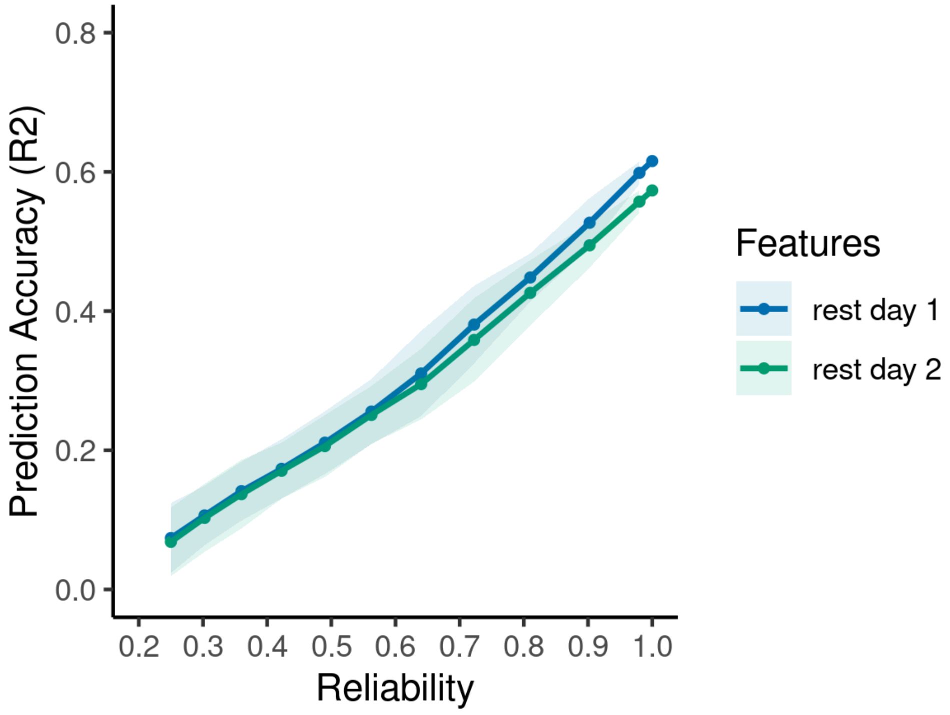

Supplementary Figure 6. Influence of feature reliability (functional connectivity) on the prediction of age with decreasing reliability. The x-axis represents the reliability of age, starting with empirically measured age at ICC ≈ 1.0, followed by age with reduced reliability through simulations as in the main text. Age prediction from functional connectivity was calculated on day 1 and 2 rsfMRI acquisition separately. Both days had 13 minutes and 22 seconds of data. Solid lines represent the mean prediction accuracy across all 100 simulated datasets in each reliability band, shaded areas represent 2 standard deviations in prediction accuracies.

Sensitivity analyses - prediction of behaviour

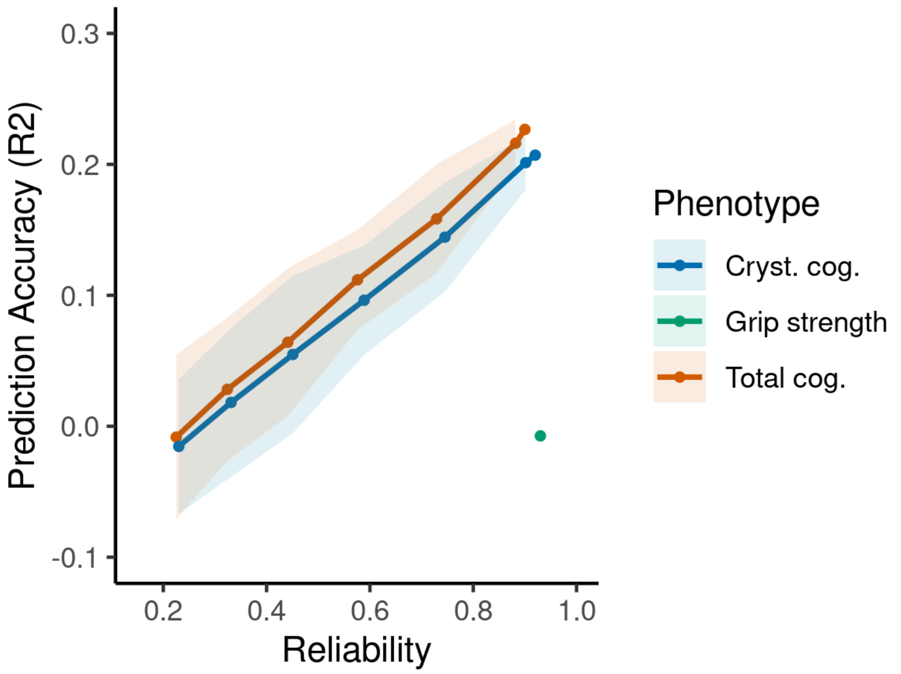

Supplementary Figure 7. Prediction of selected phenotypes with feature-wise confound (age, sex) regression. Grip strength could not be predicted when confounds were regressed. Solid lines represent the mean prediction accuracy across all 100 simulated datasets in each reliability band, shaded areas represent 2 standard deviations in prediction accuracies.

Sensitivity analyses - Total cognition

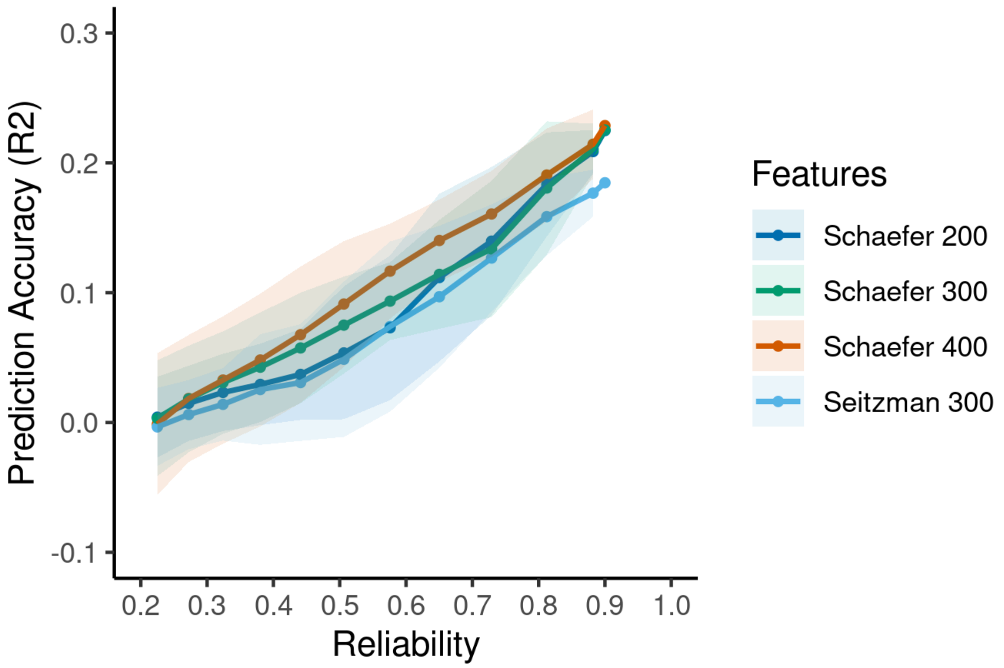

Supplementary Figure 8. Influence of feature granularity on prediction of total cognition with decreasing reliability. Solid lines represent the mean prediction accuracy across all 100 simulated datasets in each reliability band, shaded areas represent 2 standard deviations in prediction accuracies.

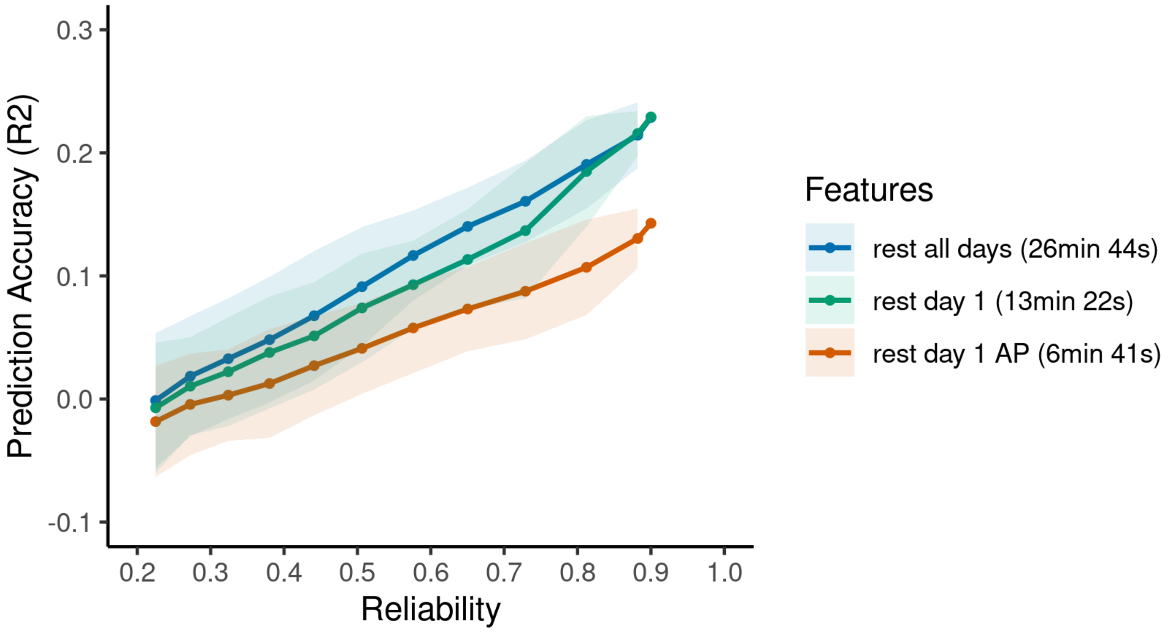

Supplementary Figure 9. Influence of feature reliability (functional connectivity) on prediction of total cognition with decreasing reliability. The x-axis represents the reliability of the NIH Toolbox total cognition composite score, starting at ICC = 0.9 based on Heaton et al. (2014), followed by total cognition with reduced reliability through simulations as in the main text. Feature reliability (represented by colours) was manipulated by reducing the amount of rsfMRI time course used to calculate functional connectivity, from the average of all four sessions collected on both days used in all main analyses to an average of 13 minutes collected on the first day only and finally, connectivity calculated from a single 6-minute session collected on the first day in the anterior-to-posterior direction (same as only session in the UKB). Solid lines represent the mean prediction accuracy across all 100 simulated datasets in each reliability band, shaded areas represent 2 standard deviations in prediction accuracies.

Phenotype prediction supplementary figures

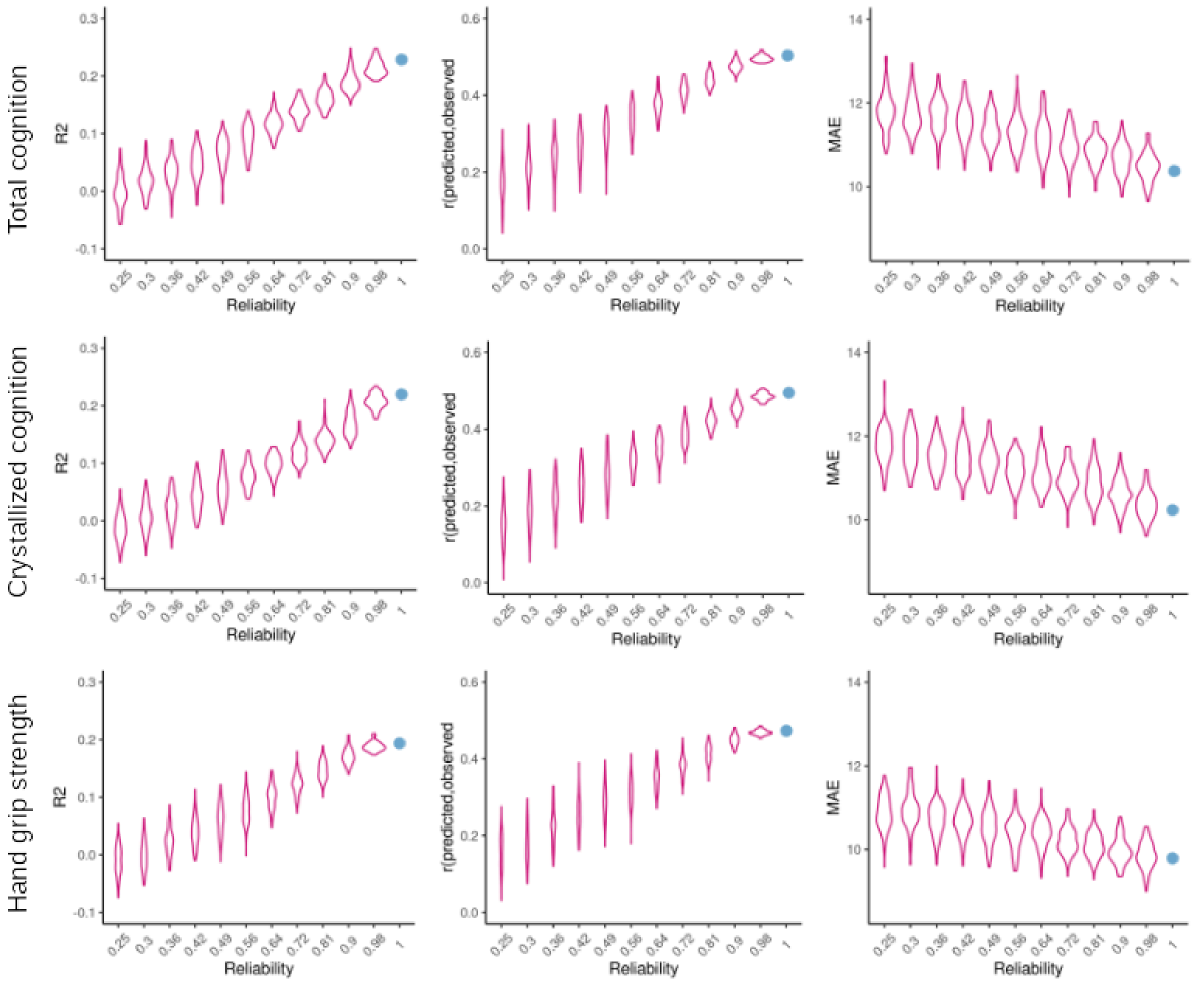

Supplementary Figure 10. R2, Mean absolute error (MAE) and correlation between observed and predicted targets for each target behaviours separately. Each violin plot summarises the accuracy of predicting 100 simulated datasets within each reliability band.

Uncorrected results for Figure 1

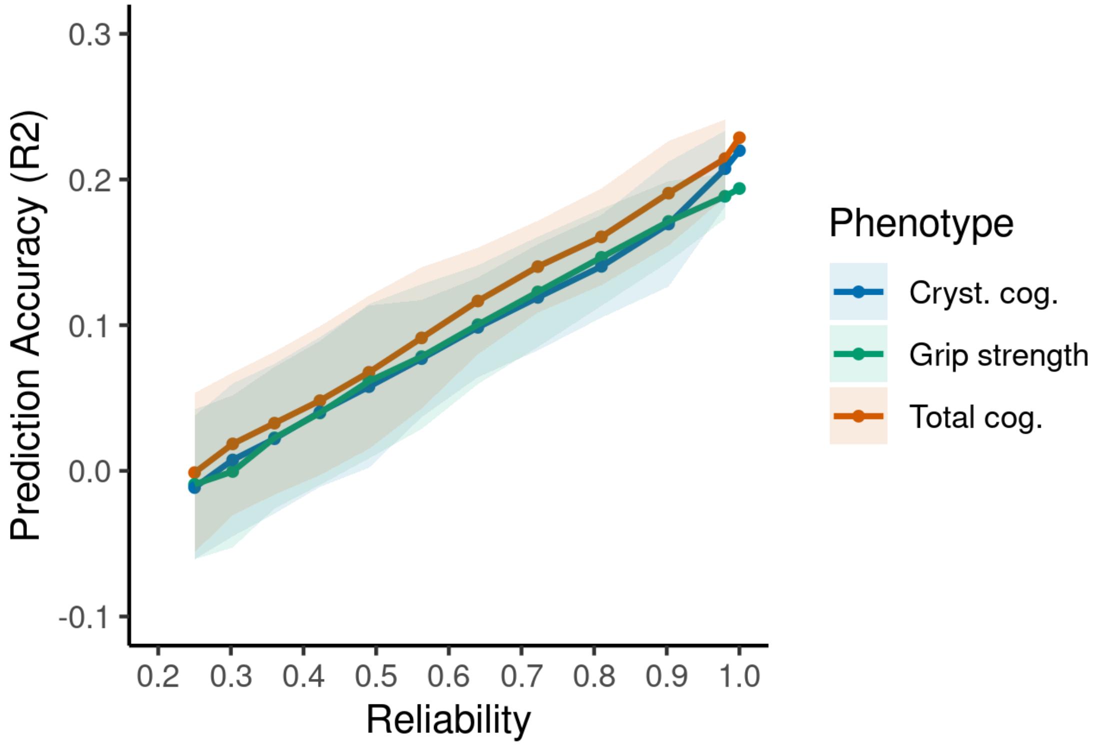

Supplementary Figure 11. Impact of reducing the correlation between original and simulated target scores (reflecting reduced reliability) on accuracy in prediction of total cognition composite score, crystallised cognition composite score and grip strength. Solid lines represent the mean across all 100 simulated datasets in each correlation band, shaded areas represent 2 standard deviations in prediction accuracies.

HCP young adult dataset predicted behaviours and respective reliability

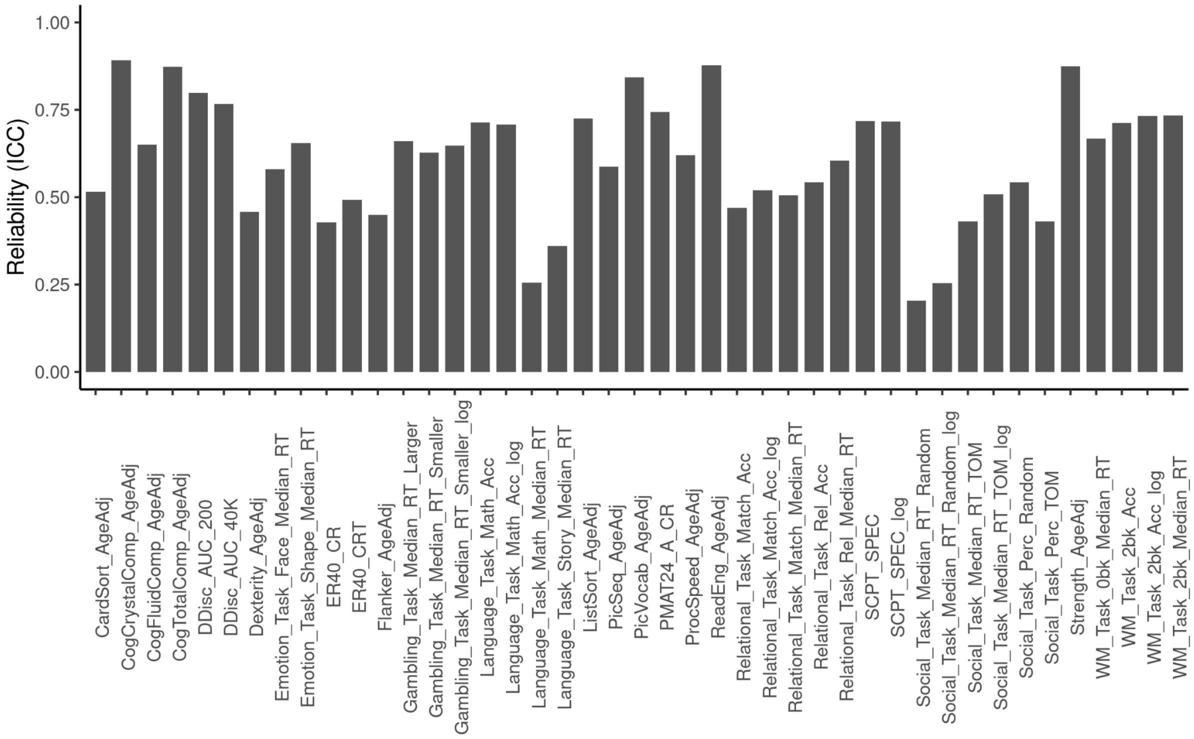

Supplementary Figure 12. Reliability of all behaviours predicted in the HCP-YA dataset

UKB predicted behaviours and respective reliability

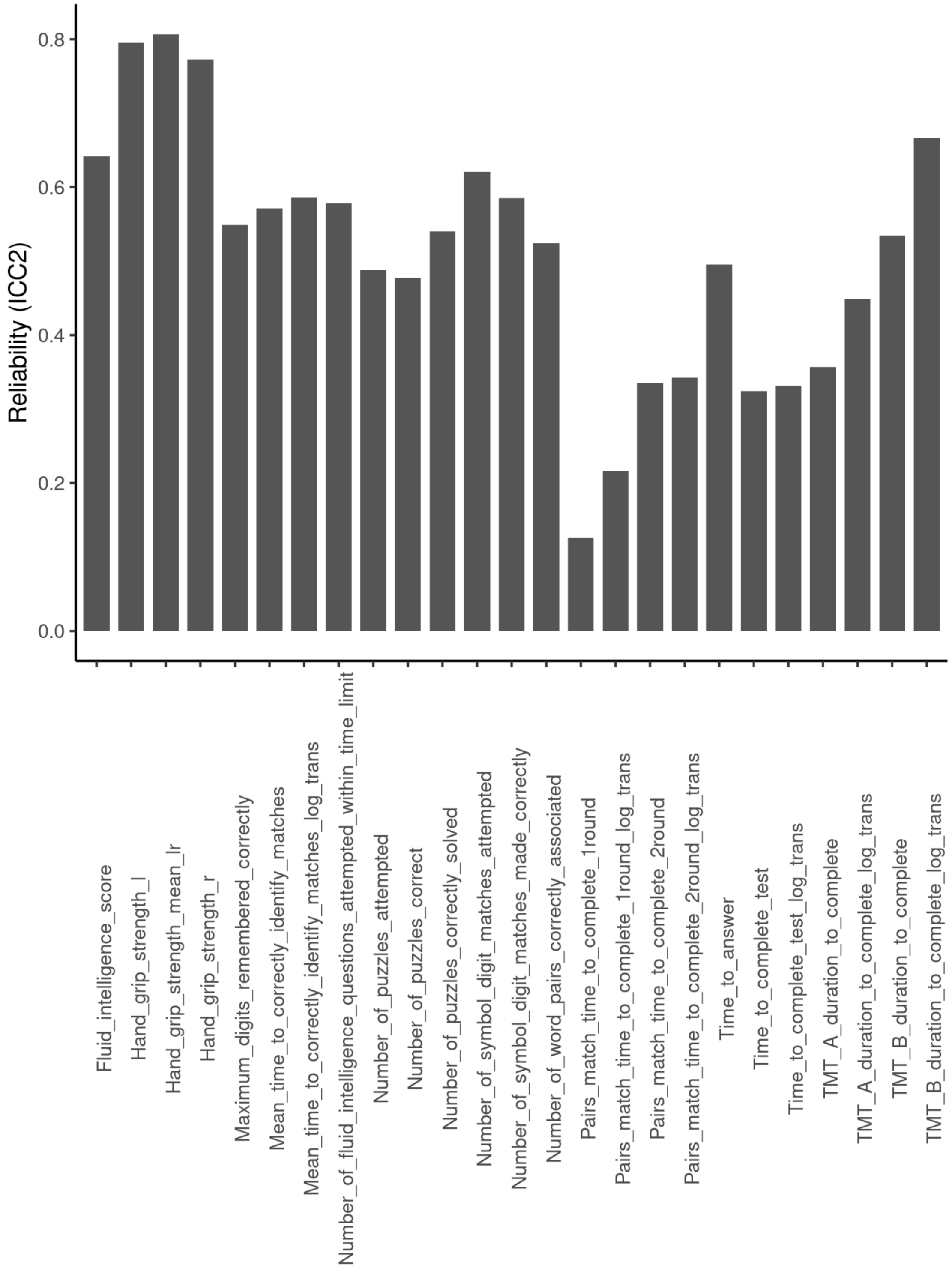

Supplementary Figure 13. Reliability of all behaviours predicted in the UKB dataset

Association between prediction accuracy and reliability in the ABCD dataset

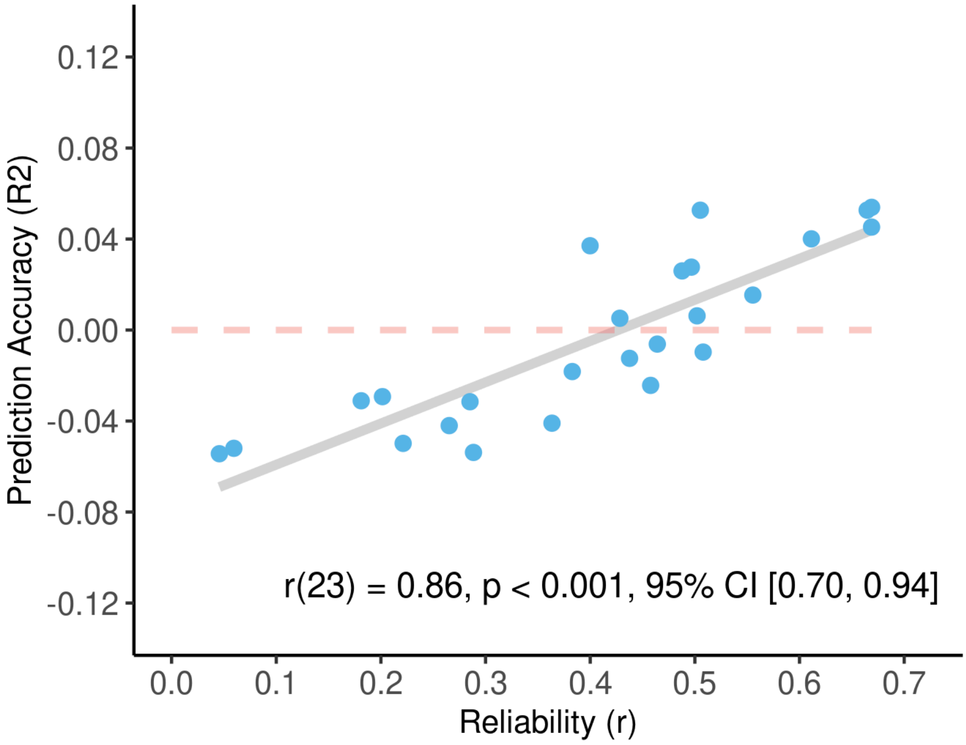

Supplementary Figure 14. Association between reliability and prediction accuracy. Each data point represents one of 25 behavioural assessments in the ABCD. As measurements were collected from participants aged 9-10 at baseline and 11-12 at follow-up (mean retest interval = 23 months), reliability is calculated with test-retest (Pearson) correlation and not ICC. In developmental contexts, unlike ICC, correlation is robust to systematic age-related changes as it is not penalised by differences in means between baseline and follow-up data and different development rates across participants.

Prediction of cognitive flexibility measured trail-making task in UKB

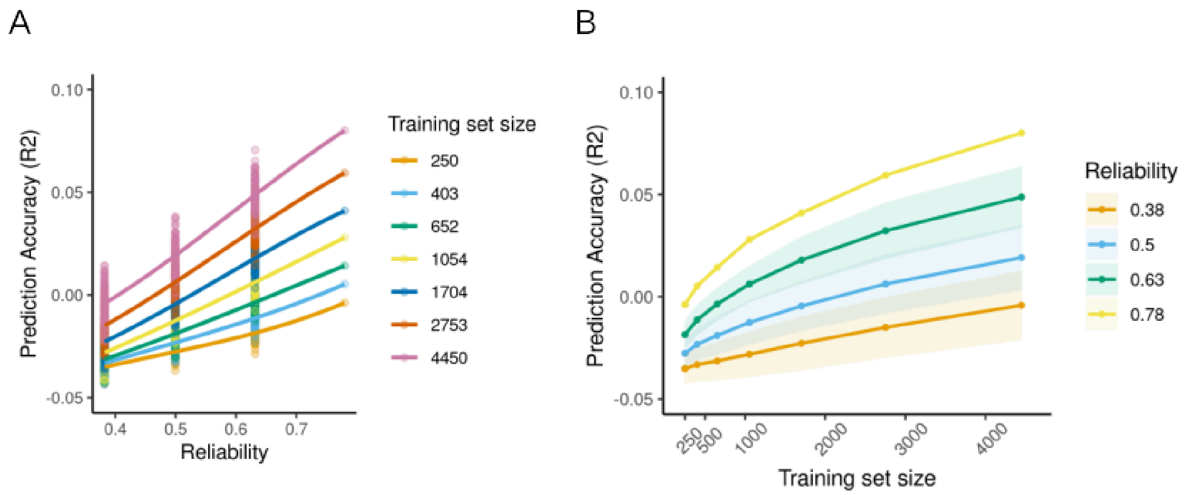

Supplementary Figure 15. Prediction and subsampling in UKB. (A) Impact of training set size on original and simulated cognitive flexibility with reduced reliability. Panel (B) Impact of training set size on prediction accuracy in empirical and simulated data with varying levels of reliability. Solid lines represent the mean across all 100 simulated datasets in each correlation band and shaded areas represent 2 standard deviations in prediction accuracies. Results were fitted with an exponential function for illustration purposes and adjusted for the TMT task's reliability (ICC = 0.775) estimated in independent data by Fawns-Ritchie et al. (2020).

Learning curve results for empirical behaviours

Supplementary Table 1

Prediction accuracy (R2) of empirical behaviours across varying training set sizes

Behaviour	Training Set Size						
	250	403	652	1054	1704	2753	4450
Fluid Intelligence	-0.02	-0.014	-0.006	0.004	0.008	0.018	0.029
Associative Learning (SDST)	-0.001	0.009	0.02	0.033	0.043	0.056	0.068
Cognitive Flexibility (TMT B)	-0.002	0.007	0.0198	0.03	0.042	0.056	0.069
Hand Grip Strength (mean)	0.127	0.16	0.197	0.24	0.279	0.313	0.344
Age	0.107	0.149	0.189	0.228	0.272	0.316	0.354

Improvement in prediction accuracy with increased training set size

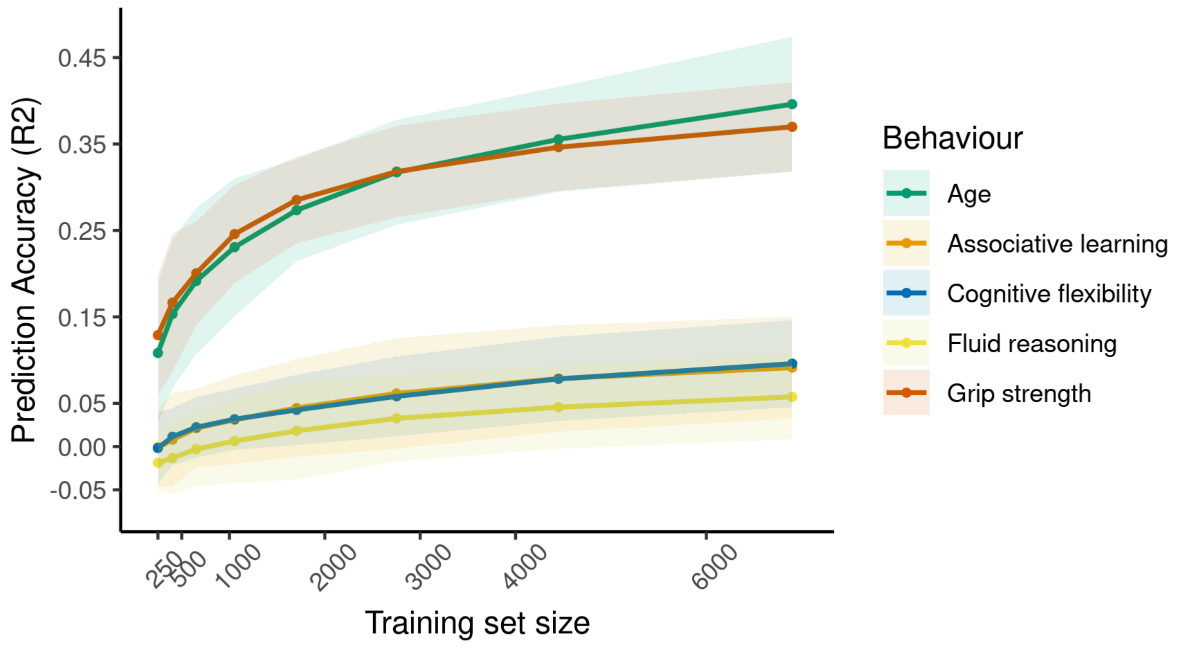

Supplementary Figure 16. Impact of training set size on prediction accuracy of empirical behaviours. Solid lines represent the mean accuracy across 100 subsamples and shaded areas represent 2 standard deviations in prediction accuracy. Abbreviations: SDST, Symbol Digit SubstitutionTest; TMT-B, Trail making task part B.

Distribution of test-retest correlations across HCP-YA, UK and ABCD datasets

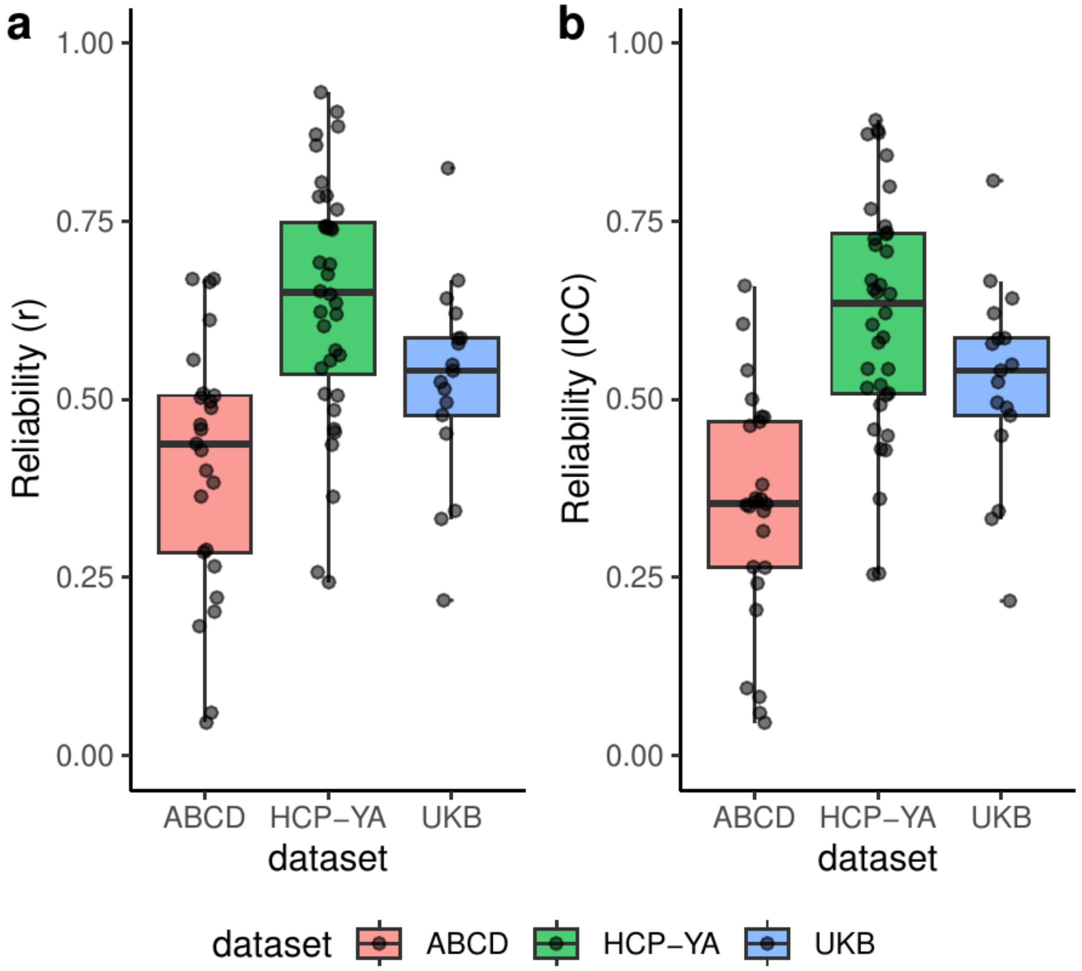

Supplementary Figure 17. Boxplots of test-retest correlations (A) and ICC (B) of all measures used for assessing the association between prediction accuracy and reliability in Figure 2 of the main text. Overall median for test-retest correlations = 0.55; and ICC = 0.51. Both retest correlations and ICC are displayed as for some datasets, such as the ABCD, test-retest correlations may be more appropriate as systematic error coming from different rates of development across participants is not penalised.

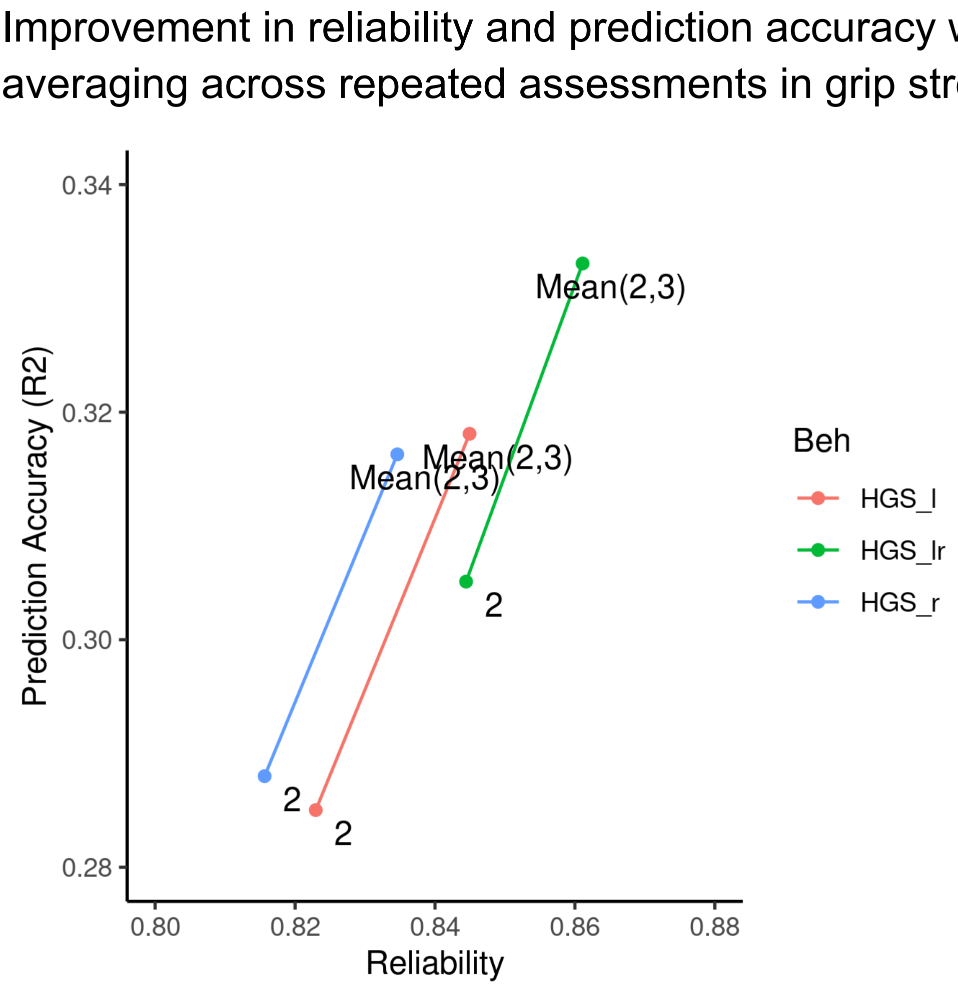

Supplementary Figure 18. Impact of increasing reliability by averaging in UKB hand grip strength prediction. Numbers denote UKB time points. Neuroimaging was collected at time point 2. Grip strength was averaged over time points 2 (neuroimaging baseline) and 3 (neuroimaging follow-up) to maximise the number of subjects. Reliability was calculated as Pearson test-retest correlation between time point 0 (baseline) and time point 2 as well as the average of 2 and 3. HGS_lr stands for the average of measurement over left and right hands. Abbreviations; HGS: Hand grip strength.

Supplementary methods

Comparison of acquisition and preprocessing parameters across datasets

Supplementary Table 2

Acquisition and preprocessing of datasets

Parameter	Dataset		
	HCP-A	HCP-YA	UKB
Scanner	Siemens Prisma 3T	Siemens 'Connectom Skyra' 3T	Siemens Skyra 3T
Resting-state sessions	4 runs across 2 days with AP and PA phase encoding on each day	4 runs across 2 days with LR and RL phase encoding on each day	a single run acquired in AP direction
acquisition time	488 frames per run (26 min total)	1200 frames per run (58 min total)	490 volumes (6 min)
TR/TE	800/37 ms	720/33 ms	735/39 ms
Acquisition sites	4	1	4
gradient distortion correction	yes	yes	yes
intensity normalisation	yes	yes	yes
motion correction	yes	yes	yes
normalisation to MNI	yes	yes	yes
artefact removal	ICA-FIX	ICA-FIX	ICA-FIX
temporal filtering	bandpass filtered at 0.01 – 0.1 Hz	bandpass filtered at 0.01 – 0.1 Hz	highpass filtering
denoising	WM+CSF+GS regression	WM+CSF+GS regression	no

Abbreviations; AP: anterior-to-posterior; PA: posterior-to-anterior; LR: left-to-right; RL right-to-left all refer to phase encoding directions

The exact sample size for predicted behaviours in HCP-A

Supplementary Table 3

Description of sample for behavioural prediction in HCP A

Behaviour	Sample (Female)	Ages
Age (in months)	647 (351)	36-86
Total cognition (age-adjusted)	550 (308)	36-86
Crystalised cognition (age-adjusted)	549 (308)	36-86
Hand grip strength (dominant hand)	551 (306)	36-86

Behaviours selected for prediction in HCP-YA dataset

Supplementary Table 4

Predicted behaviours in HCP-YA

Behaviour
PicSeq_AgeAdj
CardSort_AgeAdj
Flanker_AgeAdj
PMAT24_A_CR
ReadEng_AgeAdj
PicVocab_AgeAdj
ProcSpeed_AgeAdj
DDisc_AUC_200
DDisc_AUC_40K
ListSort_AgeAdj
SCPT_SPEC

CogFluidComp_AgeAdj

CogTotalComp_AgeAdj

CogCrystalComp_AgeAdj

ER40_CR

ER40_CRT

Strength_AgeAdj

Dexterity_AgeAdj

WM_Task_2bk_Acc

WM_Task_2bk_Median_RT

WM_Task_0bk_Median_RT

Language_Task_Math_Acc

Language_Task_Math_Median_RT

Language_Task_Story_Median_RT

Social_Task_Perc_Random

Social_Task_Perc_TOM

Social_Task_Median_RT_Random

Social_Task_Median_RT_TOM

Relational_Task_Match_Acc

Relational_Task_Match_Median_RT

Relational_Task_Rel_Acc

Relational_Task_Rel_Median_RT

Emotion_Task_Face_Median_RT

Emotion_Task_Shape_Median_RT

Gambling_Task_Median_RT_Larger

Gambling_Task_Median_RT_Smaller

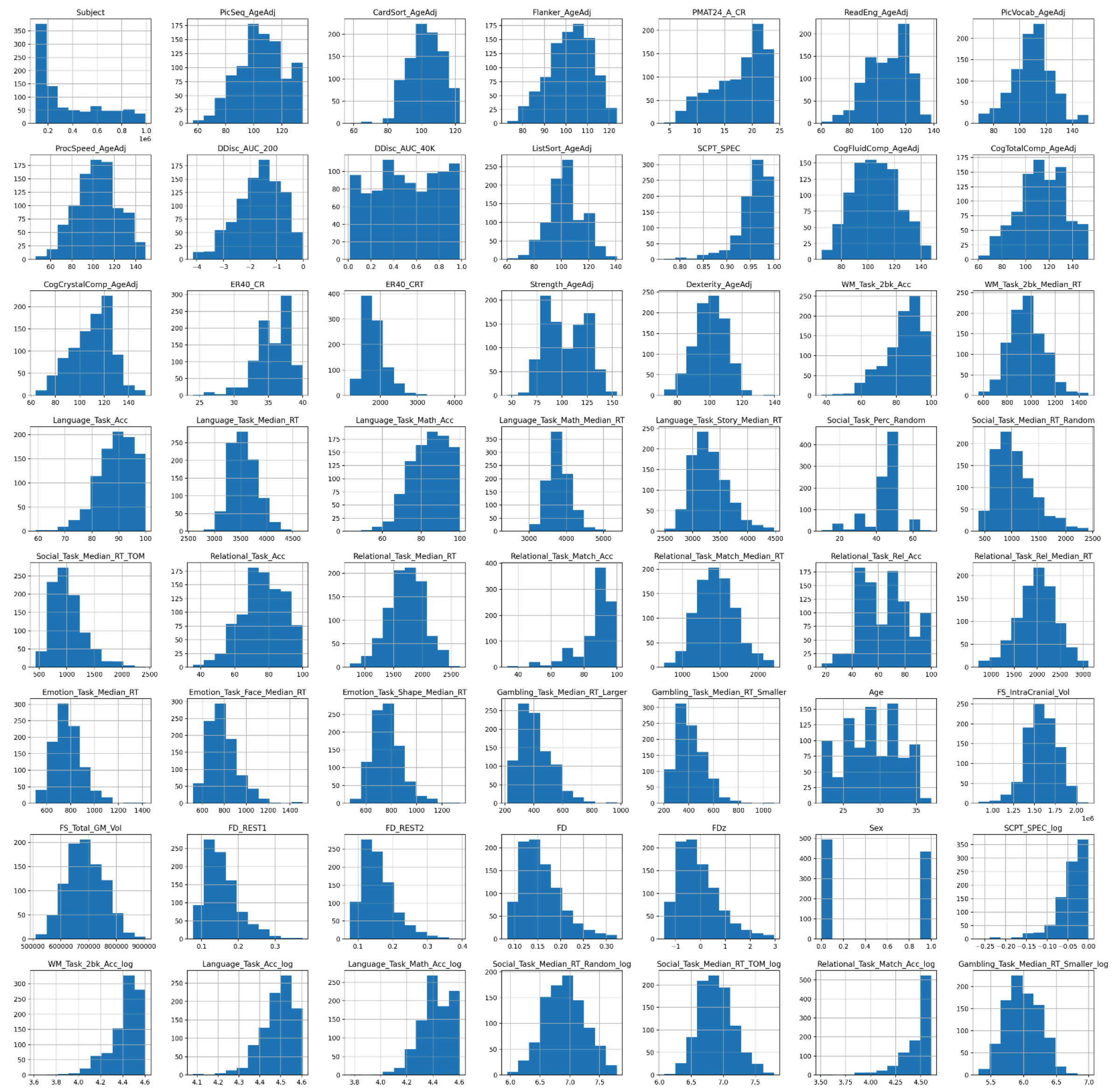

Supplementary Figure 19. Distribution of all predicted behaviours HCP-YA

Excluded fields for UKB sample

This section details excluded and included fields when parsing UKB subjects. Subjects with a history of neurological disease, as reported in (Kweon et al., 2022) and additionally subjects with sleep apnoea were excluded.

Supplementary Table 5

Excluded fields in UKB participants

Excluded ICD codes:	'G473', 'F00', 'F01', 'F02', 'F03', 'G30', 'G20' ,'G21', 'G23', 'G31', 'G32', 'G610', 'G35', 'G37', 'I63', 'G463', 'G464', 'I64', 'I694', 'C70', 'C71', 'D33', 'I60', 'I61', 'I62', 'I691', 'I692', 'I693', 'G060', 'G07', 'I671', 'Q282', 'Q283', 'G80', 'A521', 'A504', 'I64', 'A83', 'A86', 'B011', 'B020', 'B262', 'A85', 'B004', 'B582', 'A84', 'B050', 'B941', 'G04', 'A321', 'G05', 'G40', 'F803', 'S07', 'T040', 'A80', 'A81', 'A82', 'A83', 'A84', 'A85', 'A86', 'A87', 'A88', 'A89', 'G45', 'C70', 'C793', 'D32', 'D33', 'G03', 'A170', ‘A171', 'A203', 'G01', 'G02', 'G00', 'G07', 'G122', 'Q05', 'Q760', 'P100', 'I60', 'S066', 'P103', 'G45', 'F'
Self-reported illness code (Data-Coding 6):	1123, 1263, 1262, 1258, 1256, 1261, 1397, 1081, 1032, 1491, 1245, 1425, 1433, 1246, 1264, 1266, 1244, 1583, 1031, 1659, 1247, 1259, 1240, 1524, 1083, 1086, 1082

Behaviours selected for prediction in UKB dataset

Supplementary Table 6

Predicted behaviours in UKB

Behaviour	Data-field
Pairs_match_time_to_complete_1st_round-2.0	400
Pairs_match_time_to_complete_2nd_round-2.0	400
Maximum_digits_remembered_correctly-2.0	4282
Time_to_complete_test-2.0	4285
Time_to_answer-2.0	4288
TMT_A_duration_to_complete-2.0	6348

TMT_B_duration_to_complete-2.0	6350
Number_of_puzzles_correctly_solved-2.0	21004
Number_of_puzzles_attempted-2.0	6383
Fluid_intelligence_score-2.0	20016
Mean_time_to_correctly_identify_matches-2.0	20023
Number_of_fluid_intelligence_questions_attempted_within_time_limit-2.0	20128
Number_of_word_pairs_correctly_associated-2.0	20197
Number_of_puzzles_correct-2.0	6373
Number_of_symbol_digit_matches_attempted-2.0	23323
Number_of_symbol_digit_matches_made_correctly-2.0	23324
Hand_grip_strength_mean_lr-2.0*	46, 47

*Average of Hand_grip_strength_l-2.0 and Hand_grip_strength_r-2.0

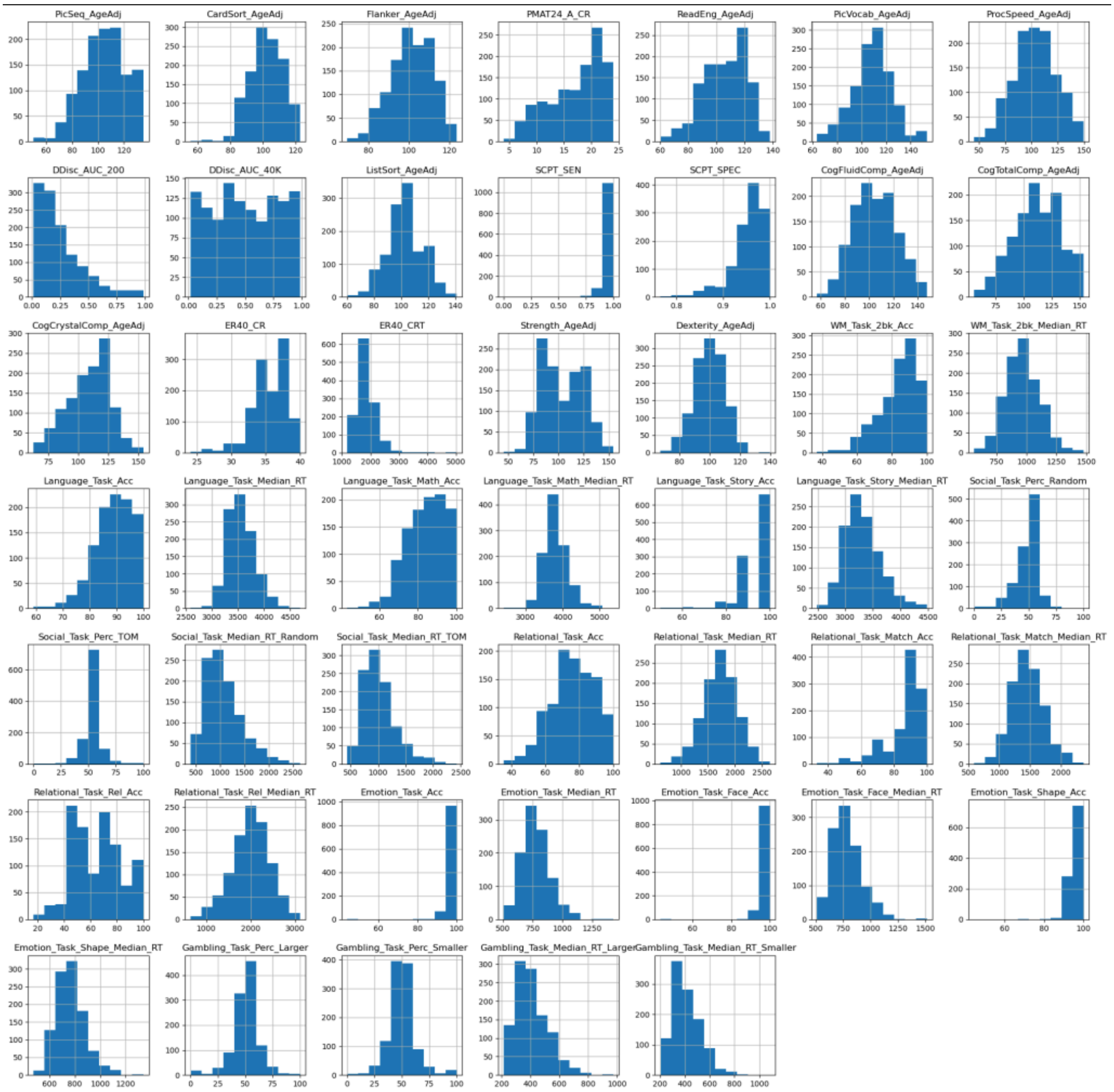

Supplementary Figure 20. Distribution of all predicted behaviours in UKB

Behaviours selected for prediction in ABCD

Supplementary Table 7

Predicted behaviours in ABCD

Behaviour
nihtbx_picvocab_fc
nihtbx_flanker_fc

nihtbx_pattern_fc

nihtbx_picture_fc

nihtbx_reading_fc

nihtbx_cryst_fc

pea_ravlt_sd_trial_vi_tc

pea_ravlt_ld_trial_vii_tc

lmt_scr_perc_correct

lmt_scr_perc_wrong

lmt_scr_avg_rt

lmt_scr_rt_correct

tfmri_mid_all_beh_srwpfb_nt

tfmri_mid_all_beh_lrwpfb_mrt

tfmri_mid_all_beh_srwpfb_mrt

tfmri_mid_all_beh_lrwpfb_nt

tfmri_sst_all_beh_total_mssrt

tfmri_nb_all_beh_c2b_rate

tfmri_nb_all_beh_c2b_mrt

tfmri_nb_all_beh_c0b_rate

tfmri_nb_all_beh_c0b_mrt

tfmri_rec_all_beh_place_dp

tfmri_rec_all_beh_negf_dp

tfmri_rec_all_beh_neutf_dp

tfmri_rec_all_beh_posf_dpr

Comparison of accuracy to computation time

Given the large number of predictions that were necessary to conduct for our simulation analyses, we first tested algorithms commonly used in the literature (Supplementary Figure

3) to find the best computation time to accuracy trade-off (measured with R2). This was not meant as an exhaustive comparison to identify the perfect pipeline. Four algorithms were tested: linear ridge regression, kernel ridge regression and two flavours of support vector regression all implemented in the Scikit learn library [version 0.24.2, (Pedregosa et al., 2011)]. A 10-fold cross-validation scheme was used to evaluate the performance of all models. Hyperparameter optimisation of the alpha regularisation parameter for ridge regression and kernel ridge regression was performed using a nested cross-validation scheme (5-fold cross-validation for kernel ridge and leave-one-out cross-validation for ridge regression) embedded with the 10-fold cross-validation. Next, two implementations of linear support vector regression were tested (sklearn.svm.LinearSVR with squared epsilon-insensitive (L2) loss function and sklearn.svm.SVR; see https://github.com/MartinGell/Prediction_Reliability for details). A heuristic was used to efficiently calculate the hyperparameter C (Helleputte, Paul, & Gramme, 2021):

$c = \frac{1}{\frac{1}{n}\sum_{i=1}^{n}\sqrt{G[i,i]}}$ where G = matrix multiplication of features and transpose of features (here functional connectivity).

Prior to training, subjects with behavioural data over +-3SD were removed and neuroimaging features were z-scored within participants (average connectomes from HCP dataset were first transformed back to r values from Fisher-z scores) to keep features consistent across algorithms as this step was required for kernel ridge regression. Within each training fold, neuroimaging features were z-scored across participants before models were trained (using Sklearn's `pipeline`).

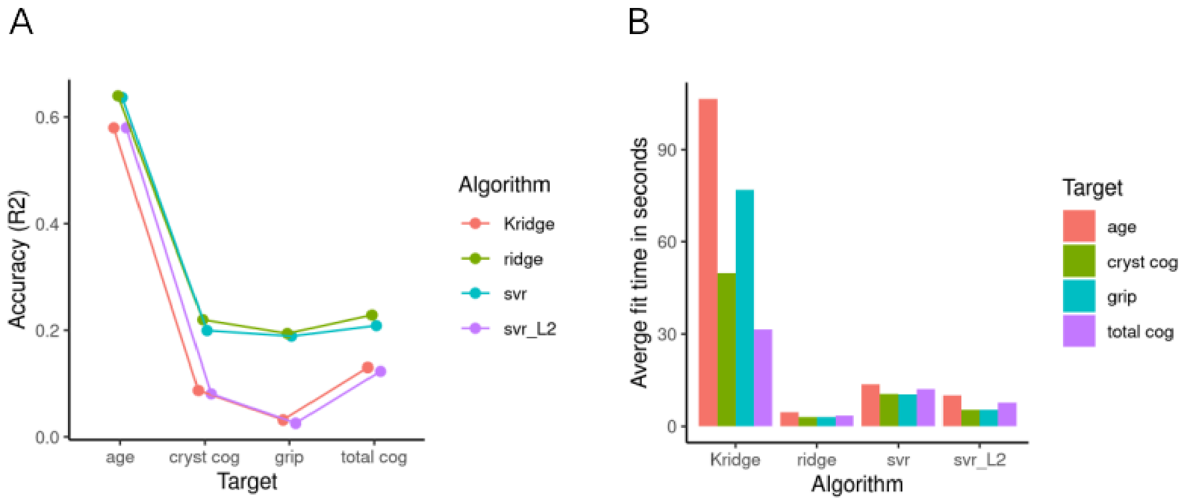

Supplementary Figure 21. Comparison of algorithms. (A) Displays prediction accuracy (R2) for all behaviours of interest predicted using different algorithms: Kridge = Kernel ridge regression, ridge = Linear ridge regression, svr = Support vector regression and svr_L2 =

support vector regression. (B) Displays the average training time of a single mode across cross-validation for all tested behaviours and algorithms.

References

Helleputte, T., Paul, J., & Gramme, P. (2021). *LiblineaR*. Retrieved from https://search.r-project.org/CRAN/refmans/LiblineaR/html/heuristicC.html

Kweon, H., Aydogan, G., Dagher, A., Bzdok, D., Ruff, C. C., Nave, G., … Koellinger, P. D. (2022). Human brain anatomy reflects separable genetic and environmental components of socioeconomic status. *Science Advances*, *8*(20), eabm2923. doi: 10.1126/sciadv.abm2923

Pedregosa, F., Varoquaux, G., Gramfort, A., Michel, V., Thirion, B., Grisel, O., … Duchesnay, É. (2011). Scikit-learn: Machine Learning in Python. *The Journal of Machine Learning Research*, *12*(null), 2825–2830.
