# Supplementary material for: How measurement noise limits the accuracy of brain-behaviour predictions
Source: Nat Commun. 2024 Dec 12;15:10678. doi: 10.1038/s41467-024-54022-6 (PMC11638260; doi:10.1038/s41467-024-54022-6)
Supplement: Supplementary file 3 — Reporting Summary [file 41467_2024_54022_MOESM3_ESM.pdf]

nature portfolio

Corresponding author(s): Martin Gell, Robert Langner

Last updated by author(s): Aug 19, 2024

Reporting Summary

Nature Portfolio wishes to improve the reproducibility of the work that we publish. This form provides structure for consistency and transparency in reporting. For further information on Nature Portfolio policies, see our Editorial Policies and the Editorial Policy Checklist.

Statistics

For all statistical analyses, confirm that the following items are present in the figure legend, table legend, main text, or Methods section.

n/a	Confirmed	
☐	☒	The exact sample size (n) for each experimental group/condition, given as a discrete number and unit of measurement
☐	☒	A statement on whether measurements were taken from distinct samples or whether the same sample was measured repeatedly
☐	☒	The statistical test(s) used AND whether they are one- or two-sided<br>*Only common tests should be described solely by name; describe more complex techniques in the Methods section.*
☐	☒	A description of all covariates tested
☐	☒	A description of any assumptions or corrections, such as tests of normality and adjustment for multiple comparisons
☐	☒	A full description of the statistical parameters including central tendency (e.g. means) or other basic estimates (e.g. regression coefficient) AND variation (e.g. standard deviation) or associated estimates of uncertainty (e.g. confidence intervals)
☐	☒	For null hypothesis testing, the test statistic (e.g. F, t, r) with confidence intervals, effect sizes, degrees of freedom and P value noted<br>*Give P values as exact values whenever suitable.*
☒	☐	For Bayesian analysis, information on the choice of priors and Markov chain Monte Carlo settings
☒	☐	For hierarchical and complex designs, identification of the appropriate level for tests and full reporting of outcomes
☐	☒	Estimates of effect sizes (e.g. Cohen's d, Pearson's r), indicating how they were calculated

Our web collection on statistics for biologists contains articles on many of the points above.

Software and code

Policy information about availability of computer code

Data collection	Data was collected by the Human Connectome Project, The Adolescent Brain Cognitive Development and UK Biobank consortia.
Data analysis	Scikit learn was used for prediction algorithms. This was wrapped up in custom code (Gell, 2024) available at: https://github.com/MartinGell/Prediction_Reliability

For manuscripts utilizing custom algorithms or software that are central to the research but not yet described in published literature, software must be made available to editors and reviewers. We strongly encourage code deposition in a community repository (e.g. GitHub). See the Nature Portfolio guidelines for submitting code & software for further information.

Data

Policy information about availability of data

All manuscripts must include a data availability statement. This statement should provide the following information, where applicable:

- Accession codes, unique identifiers, or web links for publicly available datasets
- A description of any restrictions on data availability
- For clinical datasets or third party data, please ensure that the statement adheres to our policy

This study utilised publicly available data from the UK Biobank (https://www.ukbiobank.ac.uk/enable-your-research), the HCP Young Adult (https://www.humanconnectome.org/study/hcp-young-adult), HCP Aging (https://www.humanconnectome.org/study/hcp-lifespan-aging/data-releases), and ABCD (https://nda.nih.gov/study.html?id=2313). Only researchers with an approved NDA Data Use Certification (DUC) may obtain ABCD and HCP Aging study data. Similarly, to access data from the UKB, researchers are required to comply with a data use agreement and apply for the data resource (https://www.ukbiobank.ac.uk/register-

apply/).

Research involving human participants, their data, or biological material

Policy information about studies with human participants or human data. See also policy information about sex, gender (identity/presentation), and sexual orientation and race, ethnicity and racism.

Reporting on sex and gender	Participant sex was present in all assessed datasets and was based on self-report. No sex-specific analyses were performed, instead sex was treated as a confound in prediction analyses. All non aggregated participant counts split by sex are reported in table 1.
Reporting on race, ethnicity, or other socially relevant groupings	Majority of results presented in this manuscript were from simulations. Predictions of empirical data were not corrected for socially constructed variables such as ethnicity. Instead age and sex were regressed out from functional connectivity in predictions in a cross-validation safe way.
Population characteristics	Only healthy participants were used for all analyses.
Recruitment	Not applicable (see Van Essen, 2012; Bookheimer et al., 2019; Harms et al., 2018; Sudlow et al., 2015; Casey et al., 2018).
Ethics oversight	The reanalysis of openly available data was approved by the ethics committee of the Medical Faculty at the Heinrich Heine University Düsseldorf (4039 and 2018-317-RetroDEuA). Each dataset used this study obtained ethical approval by respective ethics committee. Participants in all datasets gave informed written consent and were compensated by the respective studies and collection sites.

Note that full information on the approval of the study protocol must also be provided in the manuscript.

Field-specific reporting

Please select the one below that is the best fit for your research. If you are not sure, read the appropriate sections before making your selection.

- [x] Life sciences
- [ ] Behavioural & social sciences
- [ ] Ecological, evolutionary & environmental sciences

For a reference copy of the document with all sections, see nature.com/documents/nr-reporting-summary-flat.pdf

Life sciences study design

All studies must disclose on these points even when the disclosure is negative.

Sample size	Different samples from openly available datasets were used in order to capture various demographic groups and populations. Prediction of simulated and empirical data was performed on 647 participants from the Human Connectome dataset ageing and 5000 participants from the UK biobank dataset. Prediction of a broad range of phenotypes was done in 771 participants from the Human Connectome dataset young adult and replicated in the UK Biobank and ABCD datasets. Test-retest reliability was assessed in 46 participants of the Human Connectome young adult, 1890 subjects in the UK biobank datasets and ABCD dataset in 4133 participants. No apriori power analysis to determine required sample size were calculated as we specifically investigated the interaction between sample size, reliability and prediction accuracy. All sample sizes are presented in table 1.
Data exclusions	In the prediction analyses, we excluded all subjects with missing imaging data and excessive movement. Subjects with missing behavioural data were excluded separately for each behaviour as different subjects were missing different assessments (sample size for each behaviour is available in supplementary methods table 3). For prediction of simulated data, subjects with simulated behavioural data above and below 3 SD were removed to avoid extreme values resulting from random sampling to influence model fitting.
Replication	The reported pattern of results (reduction in prediction accuracy with reducing reliability) replicates across different behaviours (Figs. 1A and B), datasets (Figs. 1 and 3) and prediction algorithms (supplementary material).
Randomization	Since our design did not require experimental groups, no randomization was performed.
Blinding	In our design blinding was not a relevant feature, e.g. due to the lack of experimental groups

Reporting for specific materials, systems and methods

We require information from authors about some types of materials, experimental systems and methods used in many studies. Here, indicate whether each material, system or method listed is relevant to your study. If you are not sure if a list item applies to your research, read the appropriate section before selecting a response.

Materials & experimental systems

n/a	Involved in the study
☒	☐ Antibodies
☒	☐ Eukaryotic cell lines
☒	☐ Palaeontology and archaeology
☒	☐ Animals and other organisms
☒	☐ Clinical data
☒	☐ Dual use research of concern
☒	☐ Plants

Methods

n/a	Involved in the study
☒	☐ ChIP-seq
☒	☐ Flow cytometry
☐	☒ MRI-based neuroimaging

Plants

Seed stocks: n/a

Novel plant genotypes: n/a

Authentication: n/a

Magnetic resonance imaging

Experimental design

Design type: Only resting-state fMRI was used

Design specifications: not applicable

Behavioral performance measures: A wide variety of behavioural assessments were used. For simulation analyses only highly reliable data was used. See methods section for details and supplementary material for all predicted behaviours.

Acquisition

Imaging type(s): Functional and structural during pre-processing

Field strength: 3 Tesla

Sequence & imaging parameters: All imaging parameters are presented in supplementary table 5. Briefly, HCP Young adult: 2 mm (isotropic) multiband EPI, TR = 720ms + 0.7 mm (isotropic) T1W MPRAGE; HCP Aging: 2 mm (isotropic) multiband EPI, TR = 800ms + 0.8 mm (isotropic) T1W MPRAGE; UK biobank: 2.4 mm (isotropic) multiband EPI, TR = 735ms + 1mm T1W MPRAGE. ABCD: 2.4 mm (isotropic) EPI, TR = 800ms + 1.0 mm (isotropic), TR = 2500 ms MPRAGE.

Area of acquisition: Whole brain scans

Diffusion MRI ☐ Used ☒ Not used

Preprocessing

Preprocessing software: We used the HCP preprocessed data with additional denoising steps: regression of mean time courses of white matter, cerebrospinal fluid and the global signal. Next, data were linearly detrended and bandpass filtered at 0.01 – 0.1 Hz. For UK Biobank imaging we used preprocessed data available from UK Biobank (Alfaro-Almagro et al., 2018), no low-pass temporal or spatial smoothing was applied. ABCD data were detrended, demeaned and denoising using a general linear model with regressors for tissue classes and movement. The data were then bandpass filtered between 0.008 and 0.09 Hz using a 2nd order Butterworth filter. DPB respiratory motion filtering (18.582 to 25.726 breaths per minute), and censoring (frames exceeding an FD threshold of 0.2mm or failing to pass outlier detection at +/- 3 standard deviations were discarded) were then applied.

Normalization: Normalization was performed using a single nonlinear transformation.

Normalization template: Data was normalized to MNI space.

Noise and artifact removal: ICA-FIX was applied by HCP, ABCD BIDS Community Collection and UKB consortia.

Volume censoring | No volume censoring was performed.

Statistical modeling & inference

Model type and settings: Multivariate functional connectivity analysis using pearson correlation without GLM.

Effect(s) tested: Accuracy of behaviour prediction from functional connectivity.

Specify type of analysis: [x] Whole brain [ ] ROI-based [ ] Both

Statistic type for inference (See Eklund et al. 2016): Does not apply

Correction: Does not apply

Models & analysis

n/a	Involved in the study
[ ]	[x] Functional and/or effective connectivity
[x]	[ ] Graph analysis
[ ]	[x] Multivariate modeling or predictive analysis

Functional and/or effective connectivity: Pearson correlation

Multivariate modeling and predictive analysis: Linear ridge regression regression was used to predict behavioural assessments and simulated data from functional connectivity. Out-of-sample prediction accuracy was evaluated using a nested cross-validation with 10 outer folds and 5 repeats. Hyperparameter optimization (inner training folds) of the alpha regularisation parameter was done within the nested cross-validation. Accuracy was evaluated using R2 (percentage of variance explained), mean absolute error (MAE) and Pearson correlation between predicted and observed target values. No feature extraction was performed.
